# Erysipelas and Childbed Fever

**Published:** 1875-03

**Authors:** 


*Erysipelas and Child-bed Fever.* By Thomas C. Minor, M. D. Cincinnati : Robert Clarke & Co., 1874.

The object of this essay is to groove the relation between erysipelas and child-bed fever. The essay was published first in the *Cincinnati Lancet and Observer*, running through several numbers, and in this form attracted considerable attention. The author announces himself as a firm believer in the doctrine that erysipelas and child-bed fever are dependent on a common poison, and brings forward a large number of statistics and facts to support his belief. The course of the two diseases, in Cincinnati and other localites, is traced with considerable minuteness, and their intimate relation or dependence shown in several illustrative instances. Aside from the fact that a large number of valuable statistics are brought together in its pages, the monograph will repay careful reading, and will suggest to many minds several topics for consideration.

